# Effects of dietary betaine on body temperature indices, performance, metabolism, and hematological variables of dairy heifer calves during hot summer conditions

**DOI:** 10.14202/vetworld.2022.1657-1664

**Published:** 2022-07-14

**Authors:** Mohmmad Al-Qaisi, Mohamed A. Abedal-Majed, Mohannad Abuajamieh, Mufeed Alnimer, Abdur-Rahman A. Al-Fataftah, Rabie Irshaid, Hosam Titi, Anas Abdelqader

**Affiliations:** Department of Animal Production, School of Agriculture, The University of Jordan, Amman 11942, Jordan

**Keywords:** betaine, dairy calves, heat stress

## Abstract

**Background and Aim:**

Heat stress (HS) can negatively impact farm animal productivity and adversely affect animal welfare worldwide, placing a major financial burden on global livestock producers. Dietary betaine (trimethylglycine) has been known to have several biological functions, which may aid in offering beneficial effects on livestock productivity during HS conditions. However, information on the role of dietary betaine in heat-stressed dairy heifer calves is yet to be documented. Therefore, this study aimed to assess the effects of supplementing dietary betaine on body temperature indices, blood metabolites, productive performance, and complete blood count (CBC) (hematological indices) in hyperthermic dairy heifer calves.

**Materials and Methods:**

In total, 14 Holstein heifer calves (4.0 ± 0.9 months old) were individually housed and randomly allocated to one of two dietary treatments: (1) a control diet (CON; n = 7) and (2) a control diet complemented with 21 g/d of natural betaine (BET; n = 7) top-dressed once daily. The experiment lasted for 28 d, during which all animals were subjected to natural cyclic HS conditions (26.1–39.2°C; 73.2–84.0 temperature–humidity index). Rectal temperature (RT) and respiration rate (RR) were measured twice daily (0700 and 1500 h), whereas dry matter intake (DMI) was measured once daily (0800 h). In addition, blood samples (collected from the jugular vein) were analyzed for metabolites and CBC on days 7, 14, 21, and 28.

**Results:**

Relative to CON, BET supplementation was able to decrease RT on day 23 of the experiment (p = 0.04). Alternatively, RR was similar between the dietary treatments (p = 0.73). Feeding BET did not affect DMI compared with CON during HS conditions (p = 0.48). Furthermore, compared with CON, BET supplementation did not change leukocytes, neutrophils, lymphocytes, and hematocrit levels during HS conditions (p ≥ 0.17). However, a *post hoc* analysis indicated that hematocrit levels were decreased in BET-fed calves on day 7 of the study compared with CON calves during HS conditions (p = 0.05). Moreover, circulating glucose, albumin, and triglycerides were found to be similar between dietary treatments (p ≥ 0.55).

**Conclusion:**

BET supplementation slightly reduced RT and circulating hematocrit but did not affect other metrics in this HS experiment. More research into the effects of different doses of dietary BET on dairy heifer calves is needed.

## Introduction

Animals are often exposed to a myriad of environmental or physiological stressors in commercial settings. One of the most abiotic stressors that limit farm animal productivity and adversely impact animal welfare worldwide is environmental hyperthermia [1, 2]. Heat stress (HS) occurs once livestock is subjected to excessive heat from the environment (i.e., high ambient temperature and relative humidity) and metabolism, which induces a heat load, and the animals cannot lose body heat at the same rate of heat production [3, 4]. HS has caused huge financial losses to global livestock industries. For example, the dairy industry in the United States loses more than $1.5 billion each year due to the HS issue [5].

In addition, these financial losses arise from reduced milk production, diminished growth rate, declined reproductive efficiency, compromised health, and increased mortality rate [1, 4, 6]. If climate change continues to progress as expected, the aforementioned negative consequences of HS will intensify and become severe [1, 7]. Hence, HS is considered a primary risk to the global dairy sector, resulting in a food security problem, especially in developing nations [1]. Most undesirable outcomes of HS on livestock productivity stem from damaged intestinal barrier integrity [1, 2, 8]. Employing heat dissipation mechanisms in hyperthermic animals directs blood to the periphery to increase heat loss. Simultaneously, this mechanism decreases blood flow reaching the intestines, resulting in hypoxia, loss of the barrier’s functionality in intestinal epithelial cells, and oxidative stress. Furthermore, increased intestinal hyperpermeability allows various antigens, including lipopolysaccharides (LPS), to translocate into the circulation, triggering the immune system, and initiating inflammation [1, 9, 10].

Dietary betaine (trimethylglycine) has been known to have numerous important biological functions that benefit livestock productivity during HS [11, 12]. For instance, betaine works as an organic osmolyte that facilitates water molecule retention inside vital cells to avoid dehydration in situations such as HS conditions [13] and controls the osmotic pressure inside the intestinal epithelial cells. This assists in maintaining water balance and increasing intestinal cell proliferation, which would enhance nutrient digestibility and overall animal performance [14]. In addition, betaine functions as a methyl donor [15, 16] and has anti-inflammatory and antioxidant properties [16, 17]. It also aids in gut health and development [14]. Furthermore, the previous HS studies indicated that feeding betaine has increased milk and component yield (i.e., protein and fat yield) in dairy cows [12]; increased average daily gain, body weight, and dry matter intake (DMI) in buffalo heifers [13]; improved villous height. Consequently, it maintained intestinal barrier function in broiler chickens [18], decreased rectal temperature (RT) in sheep [19]; and increased feed intake, egg production, and egg mass in laying hens [20].

However, information on the role of dietary betaine in heat-stressed dairy heifer calves is yet to be clarified. Therefore, this study aimed to examine the effects of supplementing dietary betaine on body temperature indices, blood metabolites, productive performance, and complete blood count (CBC) (hematological indices) in hyperthermic dairy heifer calves.

## Materials and Methods

### Ethical approval

This trial was approved by the scientific research ethics committee of the University of Jordan regarding practices involving animals in accordance with Animal Care and Use regulations.

### Study period and location

The experiment was carried out from August 12 to September 15, 2020, at a commercial dairy farm (Dhlail area, Zarqa Governorate, Jordan) during hot season. The altitude of the study area is 581 m above the mean sea level at 32°07’51.6”N 36°17’39.9”E of latitude and longitude, respectively.

### Animal and experimental design

**Figure-1:**
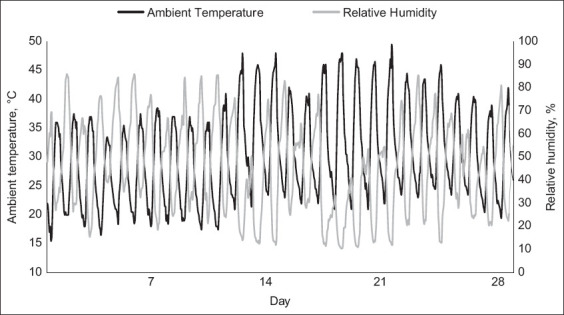
Daily ambient temperature and relative humidity throughout the experimental period.

**Figure-2:**
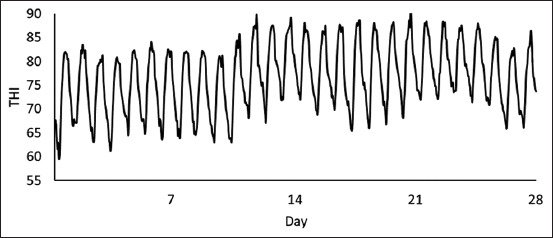
Temperature–humidity index (THI) throughout the experimental period.

In total, 14 Holstein heifer calves (4.0 ± 0.9 months old) were used in this trial and randomly allocated to one of two dietary treatments: (1) a control diet (CON; n = 7) or (2) a control diet complemented with 21 g/d of natural betaine (BET; n = 7; Betafin S4; Danisco Animal Nutrition, UK) top-dressed once daily. Calves were penned individually (1.90 × 1.90 m) in a shaded and naturally ventilated barn, allowing 6 days to acclimate to the new conditions. Following acclimation, the trial lasted for 28 days, during which all calves were subjected to cyclical natural HS situations with temperatures ranging from 26°C to 39°C (26.1°C ± 0.2°C; 55.1% ± 0.8% relative humidity; 73.2 ± 0.2 temperature–humidity index (THI) from 1900 to 1100 h, and 39.2°C ± 0.3°C; 25.5% ± 0.6% relative humidity; and 84.0 ± 0.2 THI from 1100 to 1900 h; Figures-1 and 2). Throughout the investigation, a data logger (Lascar EL-USB-2-LCD; Erie, PA, USA) recorded the ambient temperature and relative humidity every hour. All animals were under routine management practices that were followed by the farm (i.e., vaccination and deworming protocols).

### Diet and growth measurements

All calves were individually fed a total mixed ration (TMR) once a day (08:00 h) and orts were measured. The offered TMR was expected to meet the requirements of energy, protein, minerals, and vitamins [21] (Table-1). Each calf had free access to clean drinking water in a plastic bucket during the experiment. Furthermore, body weight was measured at the onset of the experimental period and the end by measuring the heart girth (HG) (chest circumference) with a common weight tape [22]. In addition, hip height (HH), hip width (HW), body length (BL), and HG were measured on days 7, 14, 21, and 28 of this study. It was obtained and recorded by the same person each time to ensure uniformity of the above-mentioned metrics.

**Table 1:** Ingredients and composition of diet^1^.

Ingredient	% of DM
Wheat straw	22.2
Alfalfa hay	44.5
Barley grains	12.0
Ground corn	4.5
Soybean meal	6.0
Wheat bran	6.0
Corn flake	2.4
Cottonseed	1.8
Limestone	0.15
Salt	0.15
Sodium bicarbonate	0.15
Vitamins and minerals mi×^2^	0.06
Chemical analysis	
Ether extract	2.2
CP	17.3
NDF	41.9
ADF	28.3
ME Mcal/kg DM	2.22

1 Values represent an average of ration nutrient summary reports collected throughout the trial. Diet dry matter averaged 52.28%.

2 The composition of each kg of mineral and vitamin premix were as follows: 6,000,000 I.U. of Vitamin A (trans-retinyl acetate), 160,000 I.U. of vitamin D3 (cholecalciferol), 1500 mg of Vitamin E (all-trac-α-tocopherol acetate), 1500 mg of Vitamin K^3^ (Menadione), 800 mg of Vitamin B1 (Thiamin), 100 mg of Vitamin B2 (Riboflavin), 30 mg of D-Pantothenate, 20 mg of Vitamin B6 (pyridoxine), 0.5 mg of Vitamin B12, 160 mg of Niacin, 4000 mg of Choline chloride, 150 g of Monocalcium Phosphate (17% Ca, 26% P), 160 g Calcium Carbonate (40% Ca), 40 g Magnesium phosphate (28% Mg, 24% P), 120 mg of Potassium iodine, 50 mg of Cobalt carbonate, 50 mg of Sodium selenite, 100 mg of Copper sulfate, 1500 mg of Ferrous sulfate, 4000 mg of Manganese oxide, 8000 mg of Zinc sulfate, 15 g DL-methionine, 5 g lysine. CP=Crude protein, NDF=Neutral detergent fiber, ADF=Acid detergent fiber, ME=Metabolizable energy, DM=Dry matter

### Body temperature measurements

RT and respiration rate (RR) were measured twice daily (0700 and 1500 h). RT was recorded using a digital thermometer (Veterinar-Thermometer SC 12, Scala Electronic GmbH, Stahnsdorf, Germany). The RR was measured by counting flank movements at 15 s intervals and multiplying by 4 to obtain breaths per min [8].

### Blood sample collection and analysis

Blood samples (4.5 mL each) were obtained from the jugular vein at 0700 h on days 7, 14, 21, and 28 of the experiment into a tube containing K_3_EDTA for plasma collection (Vacuette tube, Greiner Bio-One, Austria). Samples were centrifuged at 1000× *g* for 15 min and were then frozen at −20°C until analysis. In addition, samples for hematological indices (i.e., CBC) analysis were obtained at 0700 h on days 7, 14, 21, and 28 of the experiment and immediately submitted to the Veterinary Diagnostic Laboratory at the University of Jordan of Science and Technology (Irbid, Jordan).

According to the manufacturers’ instructions, plasma glucose, albumin, and triglyceride levels were analyzed using commercially available kits (Glucose, Spinreact, Santa Coloma, Spain; albumin and triglycerides, Agappeswiss Diagnostics, Switzerland).

### Statistical analysis

Data were statistically analyzed using SAS v.9.4 (SAS Inst. Inc., Cary, NC). DMI, body temperature measurements, blood metabolites, CBC, HH, HW, BL, HG, and feed efficiency (gain/feed) were analyzed using the MIXED procedure of SAS with a retrogressive covariance structure, and the day of the study was the repeated effect. In addition, effects of treatment, day, and treatment-by-day interaction were added to the model. In addition, final BW and ADG were analyzed using the MIXED procedure of SAS with a diagonal covariance structure. Results are stated as least-squares means and were considered different when p ≤ 0.05 and tend to differ if 0.05 < p ≤ 0.10.

## Results

### THI and body temperature variables

**Figure-3:**
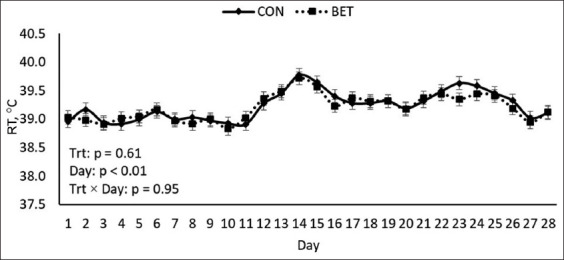
Effects of dietary betaine supplementation on rectal temperature (RT) of dairy heifer calves during hot summer conditions. Results are expressed as least squares means ± standard error of the mean.

**Figure-4:**
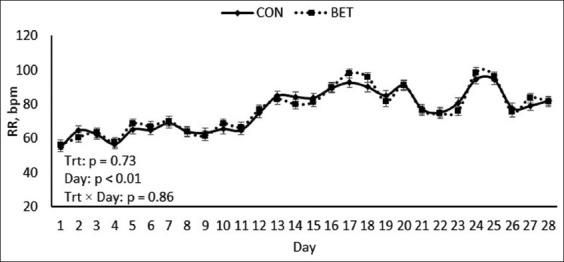
Effects of dietary betaine supplementation on respiration rate (RR) of dairy heifer calves during hot summer conditions. Results are expressed as least squares means ± standard error of the mean. Bpm = breaths per minute.

As previously stated in this study, all calves were subjected to cyclical natural HS circumstances. The maximum ambient temperature, relative humidity, and THI during HS conditions were 49.5°C, 86%, and 90.6%, respectively (Figures-1 and 2). Regardless of dietary treatments, RT and RR were noted to increase in all calves at 1500 h measurements (1.03°C and 24 breaths/min, respectively; p < 0.01; Table-2) relative to 0700 h measurements. Furthermore, the maximum RT and RR were 39.8°C on day 14 and 97 breaths/min on day 24, respectively (Figures-3 and 4). Overall, during HS conditions, RT was similar between dietary treatments (p = 0.61; Table-2, Figure-3). However, a *post hoc* analysis revealed that BET supplementation decreased RT (0.3°C; p = 0.04; Figure-3) on day 23 of the experiment relative to CON. In contrast, there were no differences in RR between the two dietary treatments (p = 0.73; Table-2, Figure-4).

**Table 2:** Effects of dietary betaine supplementation on body temperature variables of dairy heifer calves during hot summer conditions^1^.

Item	Treatment		SEM	p-value		
	
	CON	BET		Treatment	Day	Treatment×day
Rectal temperature (°C)	39.23	39.20	0.04	0.61	<0.01	0.95
0700 h	38.72	38.69	0.04	0.59	<0.01	0.99
1500 h	39.74	39.72	0.06	0.77	<0.01	0.11
Respiration rate (breaths/min)	76	77	1	0.73	<0.01	0.86
0700 h	65	64	1	0.84	<0.01	0.86
1500 h	88	89	1	0.46	<0.01	0.83

1 CON=Control diet, BET=Control diet supplemented with betaine (Betafin S4, Danisco Animal Nutrition, UK), SEM=Standard error of the mean

### Production variables

Overall, DMI was determined to be similar to dietary treatments during HS conditions (p = 0.48; Table-3). Likewise, relative to CON calves, supplementing BET did not change final body weight during HS conditions (p = 0.54; Table-3). Similarly, no differences were detected in average daily gain and feed efficiency (G: F) between dietary treatments during HS conditions (p ≥ 0.41; Table-3). Compared with CON calves, BET did not influence HH, HW, BL, and HG throughout the experiment (p ≥ 0.35; Table-3).

**Table 3:** Effects of dietary betaine supplementation on growth performance of dairy heifer calves during hot summer conditions^1^.

Item	Treatment		SEM	p-value		
	
	CON	BET		Treatment	Day	Treatment×day
DMI (kg/d)	4.7	4.5	0.2	0.48	<0.01	0.99
IBW^2^ (kg)	80.8	85.8	6	0.56	-	-
FBW^3^ (kg)	100.4	98.7	2	0.54	-	-
ADG^4^ (kg)	0.55	0.60	0.05	0.51	-	-
Gain: feed	0.12	0.13	0.01	0.41	<0.01	0.99
Hip height (cm)	94.8	94.6	0.5	0.69	<0.01	0.61
Hip width (cm)	18.6	18.9	0.2	0.35	<0.01	0.75
Body length (cm)	96.0	95.1	0.7	0.40	<0.01	0.14
Heart girth (cm)	105.3	104.5	0.8	0.48	<0.01	0.11

1 CON=control diet, BET=Control diet supplemented with betaine (Betafin S4, Danisco Animal Nutrition, UK),

2 IBW=Initial body weight,

3 FBW=Final body weight,

4 ADG=Average daily gain, SEM=Standard error of the mean

### Blood variables

**Figure-5:**
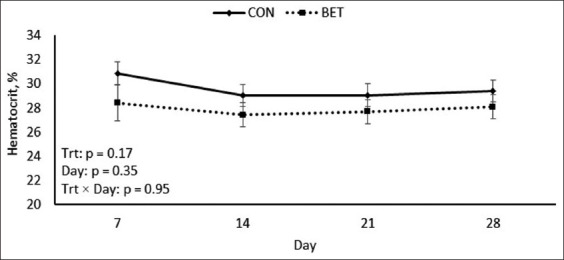
Effects of dietary betaine supplementation on circulating hematocrit of dairy heifer calves during hot summer conditions. Results are expressed as least squares means ± standard error of the mean.

Circulating leukocytes, erythrocytes, neutrophils, lymphocytes, eosinophils, monocytes, platelets, hemoglobin, and hematocrit remained unchanged between dietary treatments during HS conditions (p ≥ 0.17; Table-4). Nevertheless, a *post hoc* analysis indicated that hematocrit levels were decreased in BET-fed calves on day 7 of the study compared with CON calves during HS conditions (p = 0.05; Figure-5). In addition, no treatment differences were noted in terms of circulating glucose, albumin, and triglycerides (p ≥ 0.55; Table-5).

**Table 4:** Effects of dietary betaine supplementation on complete blood cell parameters of dairy heifer calves during hot summer conditions^1^.

Item	Treatment		SEM	p-value		
	
	CON	BET		Treatment	Day	Treatment×day
White blood cells (×103/µL)	10.05	9.57	0.64	0.62	0.73	0.82
Neutrophils (×10^3^/µL)	3.78	3.83	0.40	0.92	0.15	0.21
Red blood cells (×10^6^/µL)	9.8	9.2	0.4	0.29	<0.01	0.50
Platelets (×10^3^/µL)	564	590	24	0.47	0.13	0.72
Monocytes (×10^3^/µL)	0.043	0.045	0.016	0.93	0.69	0.41
Lymphocytes (×10^3^/µL)	5.76	4.96	0.57	0.35	<0.01	0.98
Eosinophils (×10^3^/µL)	0.23	0.30	0.06	0.37	0.04	0.13
Hemoglobin (g/dL)	9.7	9.5	0.3	0.64	<0.01	0.67
Hematocrit (%)	29.6	27.9	0.8	0.17	0.35	0.95

1 CON=Control diet, BET=Control diet supplemented with betaine (Betafin S4, Danisco Animal Nutrition, UK), SEM=Standard error of the mean

**Table 5:** Effects of dietary betaine supplementation on blood metabolites of dairy heifer calves during hot summer conditions^1^.

Item	Treatment		SEM	p-value		
	
	CON	BET		Treatment	Day	Treatment×day
Glucose (mg/dL)	79.9	78.3	2.0	0.57	0.19	0.91
Albumin (g/dL)	2.23	2.23	0.03	0.99	0.01	0.46
Triglycerides (mg/dL)	9.9	8.9	1.2	0.55	0.07	0.50

1 CON=Control diet, BET=Control diet supplemented with betaine (Betafin S4, Danisco Animal Nutrition, UK), SEM=Standard error of the mean

## Discussion

Several essential performance indicators (i.e., milk yield and composition, growth rate, reproductive efficiency, and health) are adversely affected by HS [1, 4, 6]. Hence, HS represents a significant challenge that could impose a huge financial burden on the global dairy industry [5]. HS remains a serious barrier to animal productivity during hot summer conditions, despite several management strategies (e.g., cooling systems) being devised and employed. Despite their ability to tolerate heat more than dairy cows, dairy calves are still affected by hyperthermia [4, 23]. Moreover, a possible reason for the vast majority of the harmful consequences of HS on farm animals is likely impaired intestinal health. Increased intestinal hyperpermeability allows LPS and other antigens to infiltrate the systemic circulation, thus causing inflammation [1, 9, 10, 24]. Therefore, nutritional approaches are warranted to improve animal productivity during hot summer.

Feeding betaine improves ruminant and monogastric productivity during challenging periods such as HS [11, 25, 26]. Mechanisms by which betaine exerts its positive effects in heat-stressed animals are likely through its osmoprotectant properties [16], donation of the methyl group [15], and anti-inflammatory effects [16]. Thus, we hypothesized that supplementing betaine would reduce the negative influences of HS on dairy heifer calves during hot summer conditions. Unfortunately, to the best of our knowledge, there is yet no research examining the effects of dietary betaine on dairy heifer calves during the hot summer months.

In this study, the heat load was clearly observed on all calves, as shown by the elevated body temperature variables measured (Table-2); Figures-3 and 4). Interestingly, during HS conditions, feeding betaine slightly decreased RT on day 23 of the study relative to CON calves, indicating that betaine supplementation has partially assisted calves in countering the adverse effects of hyperthermia. Similar results were observed in the previous HS studies in buffalo heifers [13], sheep [19], broiler chickens [27], and growing pigs [28]. Reduced RT is most likely related to osmoprotectant actions, which aid in restoring more cellular water, thereby assisting animals in maintaining their core body temperature [11, 13].

Environmental hyperthermia reduces DMI, which is a common response across farm animals trying to decrease metabolic heat production [2, 29]. However, this current study did not have actual thermoneutral conditions as both treatments were under cyclical HS. Therefore, we could not report a reduction in DMI caused by HS. In accordance with previous HS trials in ruminants [30, 31], DMI was not affected by dietary treatments. In contrast, other studies [12, 25, 32, 33] reported positive effects following BET supplementation on DMI. Reasons for the inconsistent effects of BET on DMI are unclear but are likely due to differences in dose, different feed compositions, HS severity, or small sample size. In the future, it would be fascinating to test BET supplementation on dairy heifer calves during HS with several dose levels and a bigger sample size.

Considering structural growth, the variables of HH, HW, BL, and HG were equal among dietary treatments, which is consistent with the findings of a previous publication [13] that found that feeding BET had no effect on BL or HG measures in buffalo heifers during the hot, humid season. Not observing differences between dietary treatments in the above-mentioned variables are predicted because DMI, ADG, and feed efficiency were similar between dietary treatments.

Interestingly, despite no treatment differences in hematocrit levels during HS conditions, hematocrit levels were numerically decreased in BET-supplemented calves relative to their CON counterparts. In addition, a *post hoc* analysis showed that hematocrit levels decreased in BET-supplemented calves on day 7 of the experiment compared with CON calves. Increasing hematocrit levels (a proxy indicator of mild dehydration) were common during HS conditions [28, 34, 35]. The previous studies have also reported decreased hematocrit following BET supplementation in heat-stressed growing pigs [28] and in laying hens under HS conditions [36]. This response likely indicates that BET can (through its osmoregulatory function) attenuate the dehydration status of heat-stressed animals. Further research must evaluate the effects of feeding BET on hematocrit percentage and dehydration during HS conditions.

Thermal stress, as previously indicated, compromises intestinal integrity and promotes inflammation by allowing antigens (e.g., LPS) to infiltrate from the lumen into systemic circulation [1, 37]. Hence, leukocyte counts were measured in this experiment as biomarkers of inflammation [38] to examine the effect of BET supplementation on those variables during hot summer conditions. Information in the literature regarding circulating leukocytes is stated during HS conditions. The previous reports have demonstrated that HS can alter the leukocyte population (increased leukocytes) in lactating dairy cows [39]. Furthermore, HS increased circulating leukocytes in sheep [40], whereas it decreased them in growing pigs [38]. In this study, leukocyte levels were not altered following BET supplementation. In contrast, providing BET to heat-stressed growing pigs decreased circulating neutrophils [28]. These discordant effects in leukocyte dynamics following BET supplementation are not fully elucidated. Further research is thus needed to define the influence of feeding BET on circulating leukocytes in heat-stressed dairy calves.

## Conclusion

All calves were subjected to chronic cyclical HS conditions as indicated by values of ambient temperature, RH, THI, and body temperature variables. Furthermore, BET supplementation somewhat reduced RT and circulating hematocrit, but it did not affect the other parameters in this HS experiment. Therefore, more research is needed in the future to assess the effects of different doses of BET supplementation on dairy heifer calves.

## Authors’ Contributions

MAQ: Designed the study, performed the methodology, investigation, statis­tical analysis, and drafted and revised the manuscript. MAA, MA, MuA, RI, and HT: Data collec­tion and carried out the study. AAA and AA: Revised the manuscript. All authors have read and approved the final manuscript.
